# Supplementary material for: Human and mouse activin genes: Divergent expression of activin A protein variants and identification of a novel heparan sulfate-binding domain in activin B
Source: PLoS One. 2020 Feb 19;15(2):e0229254. doi: 10.1371/journal.pone.0229254 (PMC7029874; doi:10.1371/journal.pone.0229254)
Supplement: S1 Fig — (PPTX) [file pone.0229254.s001.pptx]

## Slide 1
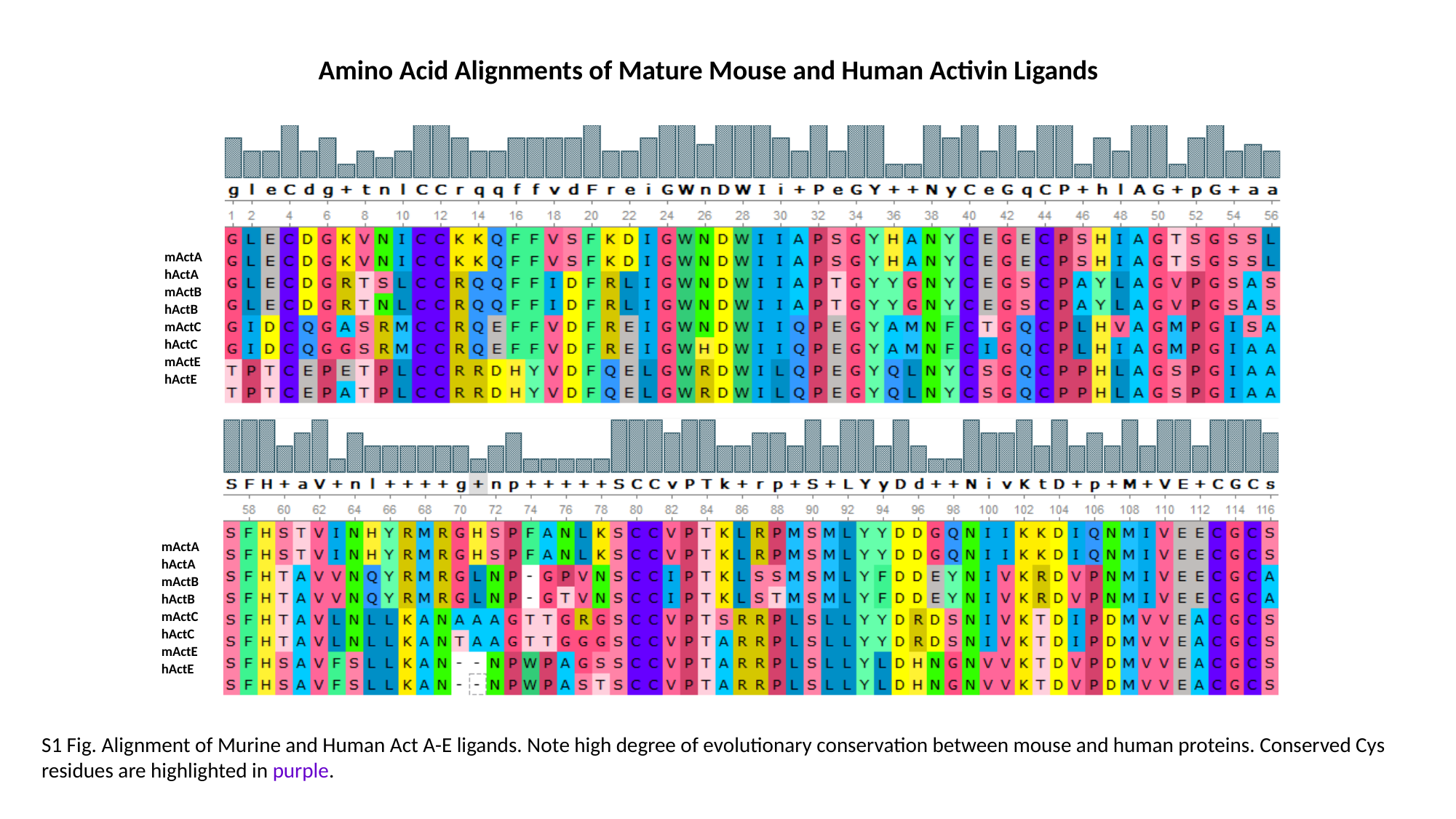

Amino Acid Alignments of Mature Mouse and Human Activin Ligands
mActA
hActA
mActB
hActB
mActC
hActC
mActE
hActE
mActA
hActA
mActB
hActB
mActC
hActC
mActE
hActE
S1 Fig. Alignment of Murine and Human Act A-E ligands. Note high degree of evolutionary conservation between mouse and human proteins. Conserved Cys residues are highlighted in purple.
