# Supplementary material for: Human and mouse activin genes: Divergent expression of activin A protein variants and identification of a novel heparan sulfate-binding domain in activin B
Source: PLoS One. 2020 Feb 19;15(2):e0229254. doi: 10.1371/journal.pone.0229254 (PMC7029874; doi:10.1371/journal.pone.0229254)
Supplement: S2 Fig — (PPTX) [file pone.0229254.s002.pptx]

## Slide 1
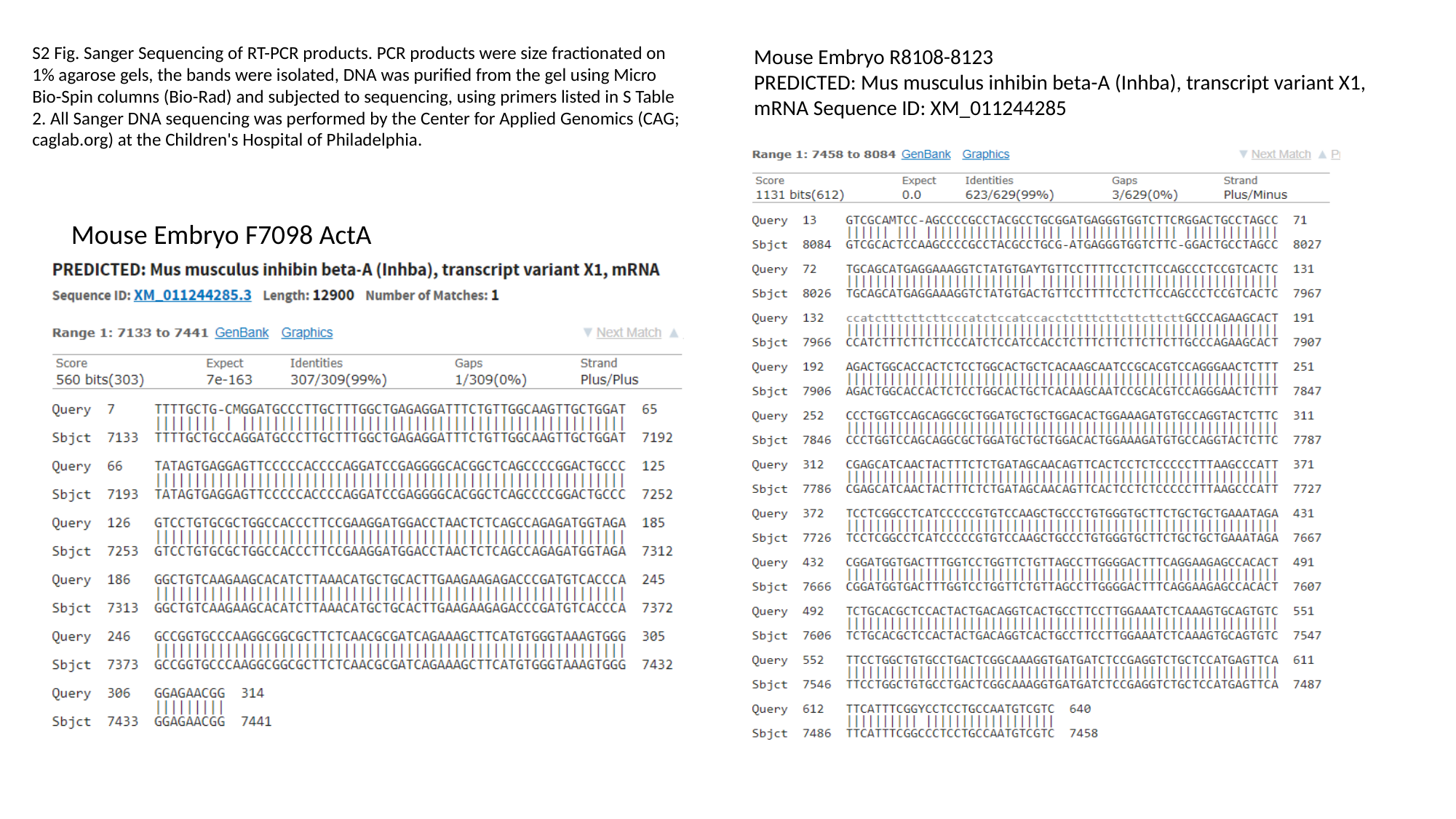

S2 Fig. Sanger Sequencing of RT-PCR products. PCR products were size fractionated on 1% agarose gels, the bands were isolated, DNA was purified from the gel using Micro Bio-Spin columns (Bio-Rad) and subjected to sequencing, using primers listed in S Table 2. All Sanger DNA sequencing was performed by the Center for Applied Genomics (CAG; caglab.org) at the Children's Hospital of Philadelphia.
Mouse Embryo R8108-8123
PREDICTED: Mus musculus inhibin beta-A (Inhba), transcript variant X1, mRNA Sequence ID: XM_011244285
Mouse Embryo F7098 ActA

## Slide 2
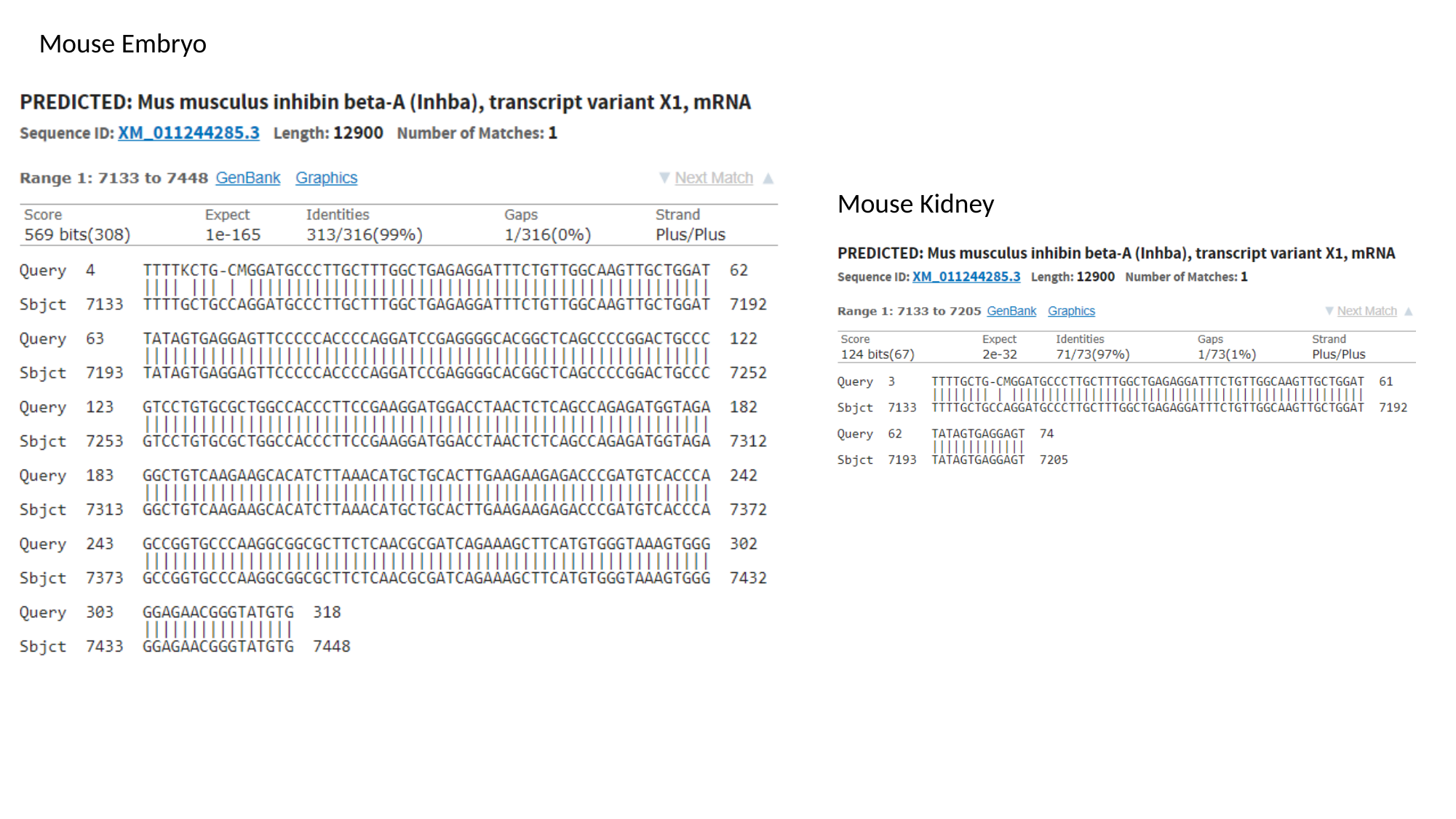

Mouse Embryo
Mouse Kidney

## Slide 3
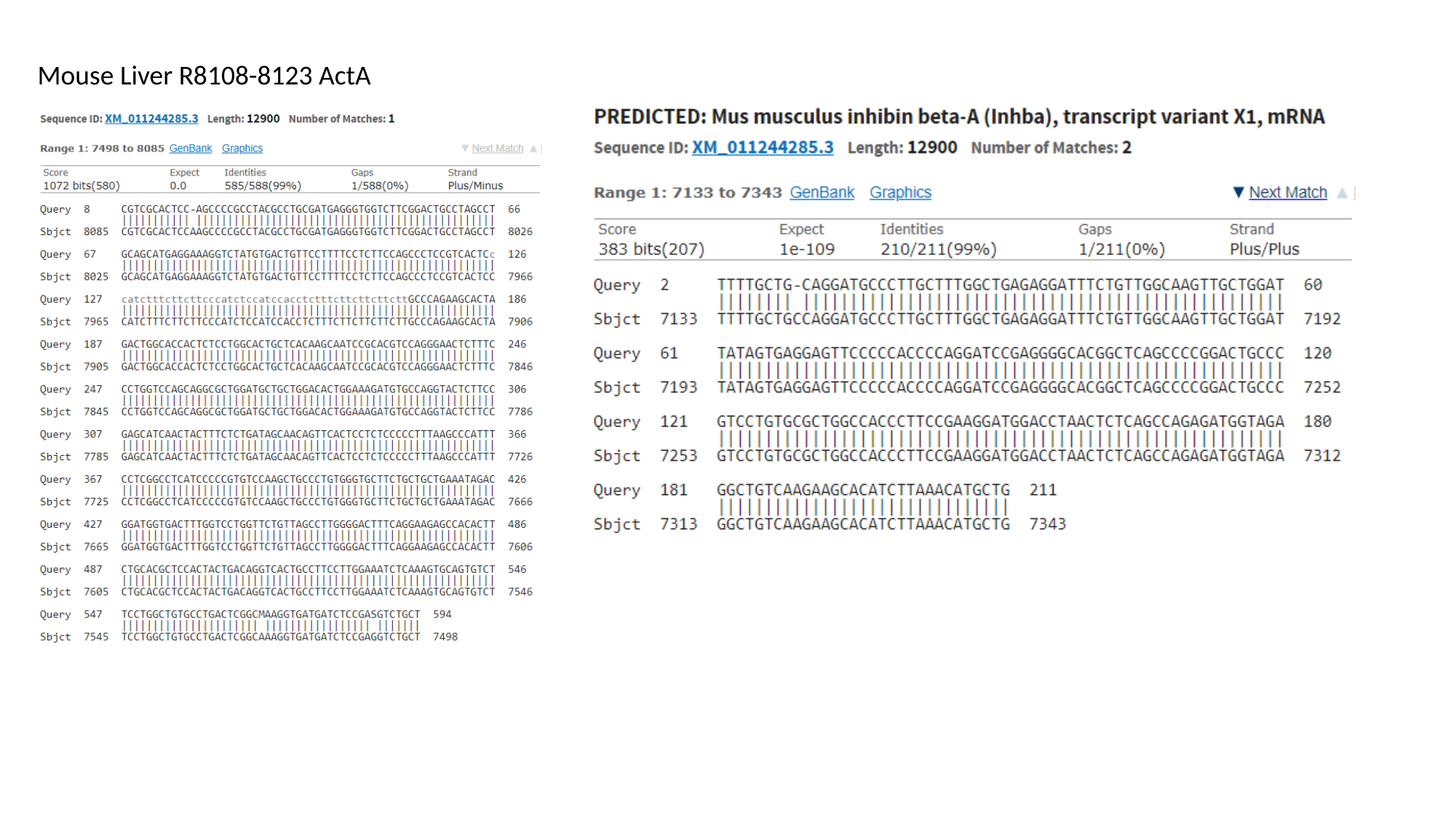

Mouse Liver R8108-8123 ActA

## Slide 4
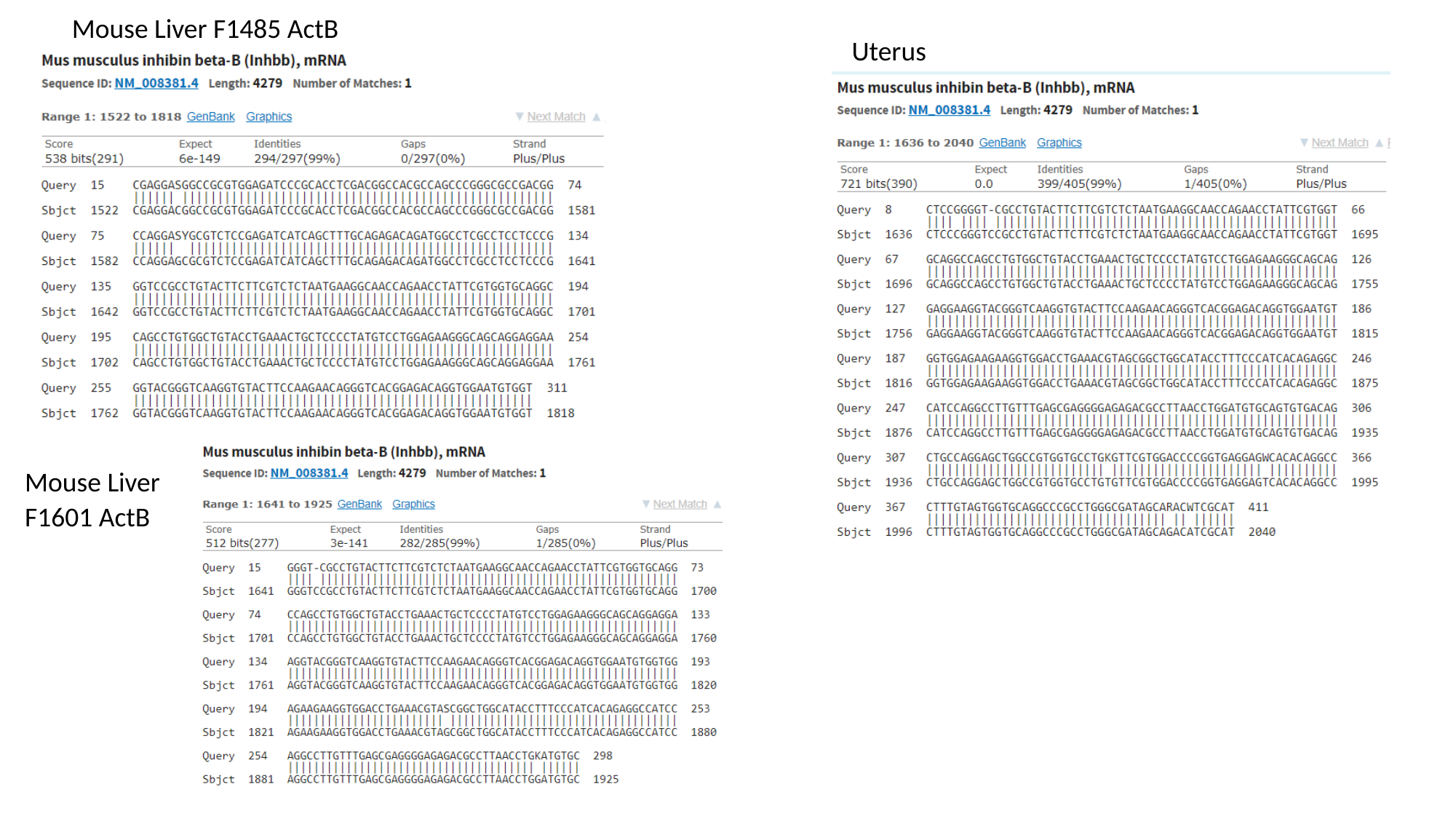

Mouse Liver F1485 ActB
Uterus
Mouse Liver F1601 ActB
