# Supplementary material for: Human and mouse activin genes: Divergent expression of activin A protein variants and identification of a novel heparan sulfate-binding domain in activin B
Source: PLoS One. 2020 Feb 19;15(2):e0229254. doi: 10.1371/journal.pone.0229254 (PMC7029874; doi:10.1371/journal.pone.0229254)
Supplement: S3 Fig — (PPTX) [file pone.0229254.s003.pptx]

## Slide 1
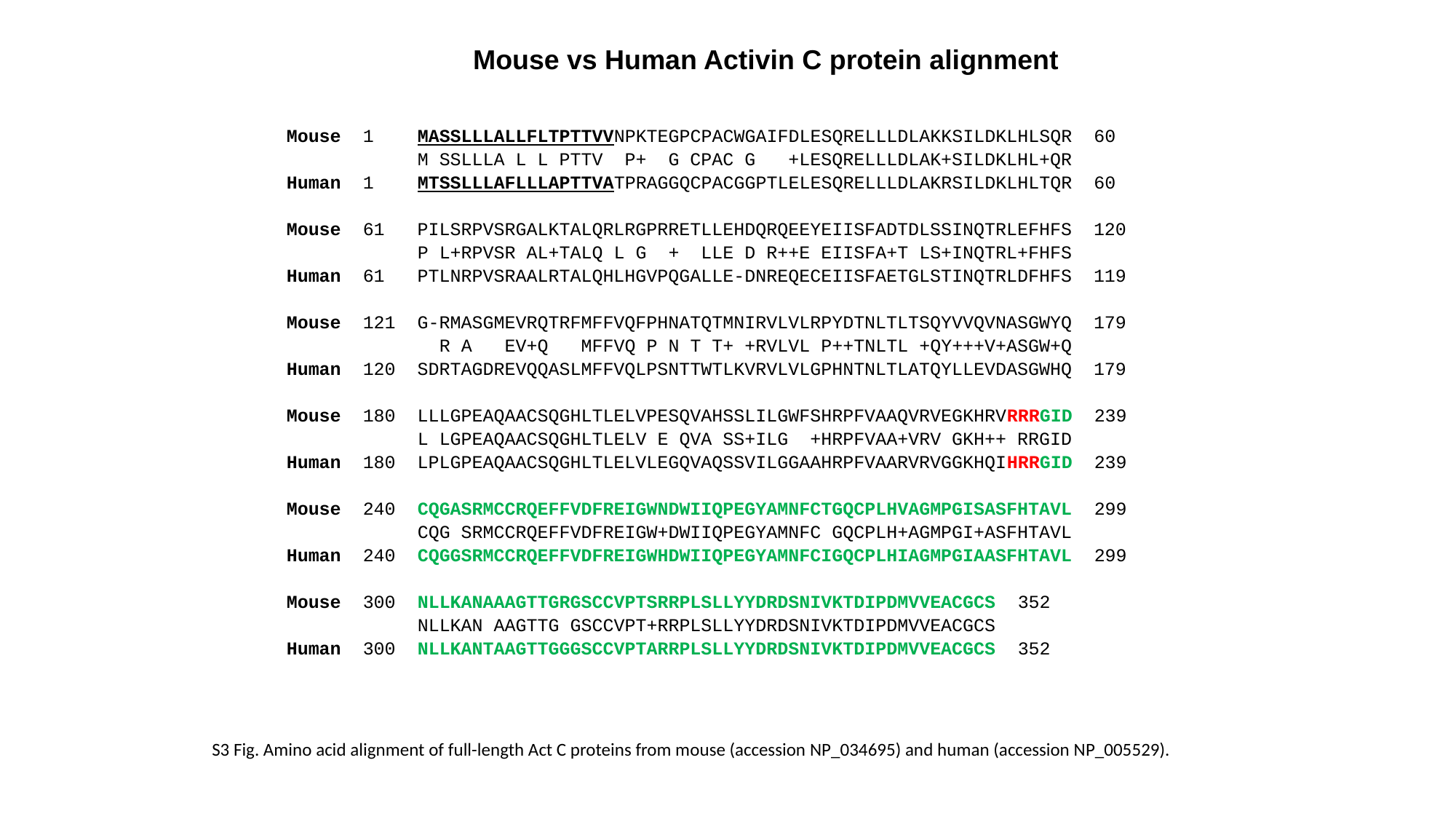

Mouse vs Human Activin C protein alignment
Mouse 1 MASSLLLALLFLTPTTVVNPKTEGPCPACWGAIFDLESQRELLLDLAKKSILDKLHLSQR 60
 M SSLLLA L L PTTV P+ G CPAC G +LESQRELLLDLAK+SILDKLHL+QR
Human 1 MTSSLLLAFLLLAPTTVATPRAGGQCPACGGPTLELESQRELLLDLAKRSILDKLHLTQR 60
Mouse 61 PILSRPVSRGALKTALQRLRGPRRETLLEHDQRQEEYEIISFADTDLSSINQTRLEFHFS 120
 P L+RPVSR AL+TALQ L G + LLE D R++E EIISFA+T LS+INQTRL+FHFS
Human 61 PTLNRPVSRAALRTALQHLHGVPQGALLE-DNREQECEIISFAETGLSTINQTRLDFHFS 119
Mouse 121 G-RMASGMEVRQTRFMFFVQFPHNATQTMNIRVLVLRPYDTNLTLTSQYVVQVNASGWYQ 179
 R A EV+Q MFFVQ P N T T+ +RVLVL P++TNLTL +QY+++V+ASGW+Q
Human 120 SDRTAGDREVQQASLMFFVQLPSNTTWTLKVRVLVLGPHNTNLTLATQYLLEVDASGWHQ 179
Mouse 180 LLLGPEAQAACSQGHLTLELVPESQVAHSSLILGWFSHRPFVAAQVRVEGKHRVRRRGID 239
 L LGPEAQAACSQGHLTLELV E QVA SS+ILG +HRPFVAA+VRV GKH++ RRGID
Human 180 LPLGPEAQAACSQGHLTLELVLEGQVAQSSVILGGAAHRPFVAARVRVGGKHQIHRRGID 239
Mouse 240 CQGASRMCCRQEFFVDFREIGWNDWIIQPEGYAMNFCTGQCPLHVAGMPGISASFHTAVL 299
 CQG SRMCCRQEFFVDFREIGW+DWIIQPEGYAMNFC GQCPLH+AGMPGI+ASFHTAVL
Human 240 CQGGSRMCCRQEFFVDFREIGWHDWIIQPEGYAMNFCIGQCPLHIAGMPGIAASFHTAVL 299
Mouse 300 NLLKANAAAGTTGRGSCCVPTSRRPLSLLYYDRDSNIVKTDIPDMVVEACGCS 352
 NLLKAN AAGTTG GSCCVPT+RRPLSLLYYDRDSNIVKTDIPDMVVEACGCS
Human 300 NLLKANTAAGTTGGGSCCVPTARRPLSLLYYDRDSNIVKTDIPDMVVEACGCS 352
S3 Fig. Amino acid alignment of full-length Act C proteins from mouse (accession NP_034695) and human (accession NP_005529).
