# Supplementary material for: Human and mouse activin genes: Divergent expression of activin A protein variants and identification of a novel heparan sulfate-binding domain in activin B
Source: PLoS One. 2020 Feb 19;15(2):e0229254. doi: 10.1371/journal.pone.0229254 (PMC7029874; doi:10.1371/journal.pone.0229254)
Supplement: S4 Fig — (PPTX) [file pone.0229254.s004.pptx]

## Slide 1
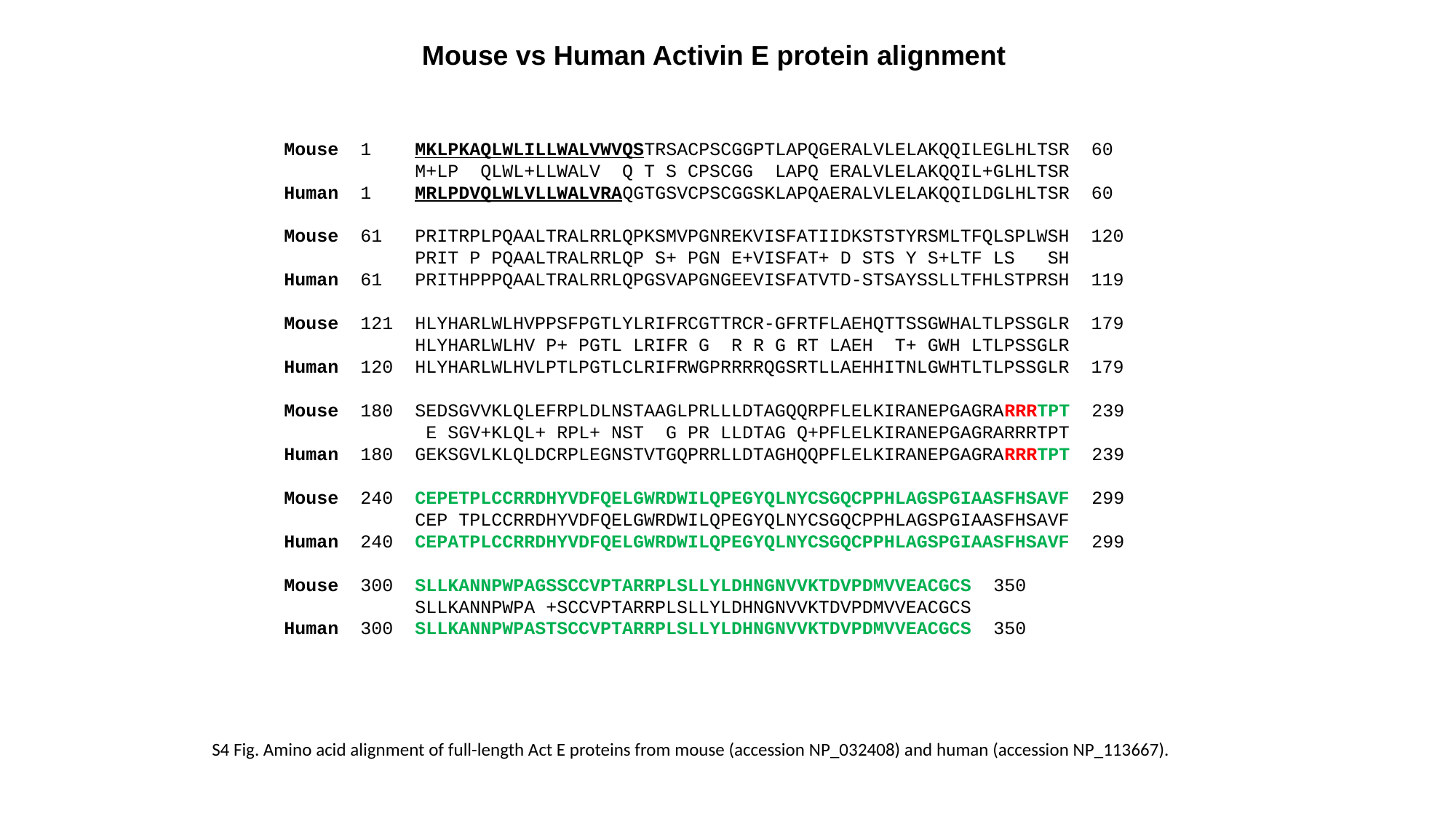

Mouse vs Human Activin E protein alignment
Mouse 1 MKLPKAQLWLILLWALVWVQSTRSACPSCGGPTLAPQGERALVLELAKQQILEGLHLTSR 60
 M+LP QLWL+LLWALV Q T S CPSCGG LAPQ ERALVLELAKQQIL+GLHLTSR
Human 1 MRLPDVQLWLVLLWALVRAQGTGSVCPSCGGSKLAPQAERALVLELAKQQILDGLHLTSR 60
Mouse 61 PRITRPLPQAALTRALRRLQPKSMVPGNREKVISFATIIDKSTSTYRSMLTFQLSPLWSH 120
 PRIT P PQAALTRALRRLQP S+ PGN E+VISFAT+ D STS Y S+LTF LS SH
Human 61 PRITHPPPQAALTRALRRLQPGSVAPGNGEEVISFATVTD-STSAYSSLLTFHLSTPRSH 119
Mouse 121 HLYHARLWLHVPPSFPGTLYLRIFRCGTTRCR-GFRTFLAEHQTTSSGWHALTLPSSGLR 179
 HLYHARLWLHV P+ PGTL LRIFR G R R G RT LAEH T+ GWH LTLPSSGLR
Human 120 HLYHARLWLHVLPTLPGTLCLRIFRWGPRRRRQGSRTLLAEHHITNLGWHTLTLPSSGLR 179
Mouse 180 SEDSGVVKLQLEFRPLDLNSTAAGLPRLLLDTAGQQRPFLELKIRANEPGAGRARRRTPT 239
 E SGV+KLQL+ RPL+ NST G PR LLDTAG Q+PFLELKIRANEPGAGRARRRTPT
Human 180 GEKSGVLKLQLDCRPLEGNSTVTGQPRRLLDTAGHQQPFLELKIRANEPGAGRARRRTPT 239
Mouse 240 CEPETPLCCRRDHYVDFQELGWRDWILQPEGYQLNYCSGQCPPHLAGSPGIAASFHSAVF 299
 CEP TPLCCRRDHYVDFQELGWRDWILQPEGYQLNYCSGQCPPHLAGSPGIAASFHSAVF
Human 240 CEPATPLCCRRDHYVDFQELGWRDWILQPEGYQLNYCSGQCPPHLAGSPGIAASFHSAVF 299
Mouse 300 SLLKANNPWPAGSSCCVPTARRPLSLLYLDHNGNVVKTDVPDMVVEACGCS 350
 SLLKANNPWPA +SCCVPTARRPLSLLYLDHNGNVVKTDVPDMVVEACGCS
Human 300 SLLKANNPWPASTSCCVPTARRPLSLLYLDHNGNVVKTDVPDMVVEACGCS 350
S4 Fig. Amino acid alignment of full-length Act E proteins from mouse (accession NP_032408) and human (accession NP_113667).
