# Supplementary material for: Human and mouse activin genes: Divergent expression of activin A protein variants and identification of a novel heparan sulfate-binding domain in activin B
Source: PLoS One. 2020 Feb 19;15(2):e0229254. doi: 10.1371/journal.pone.0229254 (PMC7029874; doi:10.1371/journal.pone.0229254)
Supplement: S5 Fig — (PPTX) [file pone.0229254.s005.pptx]

## Slide 1
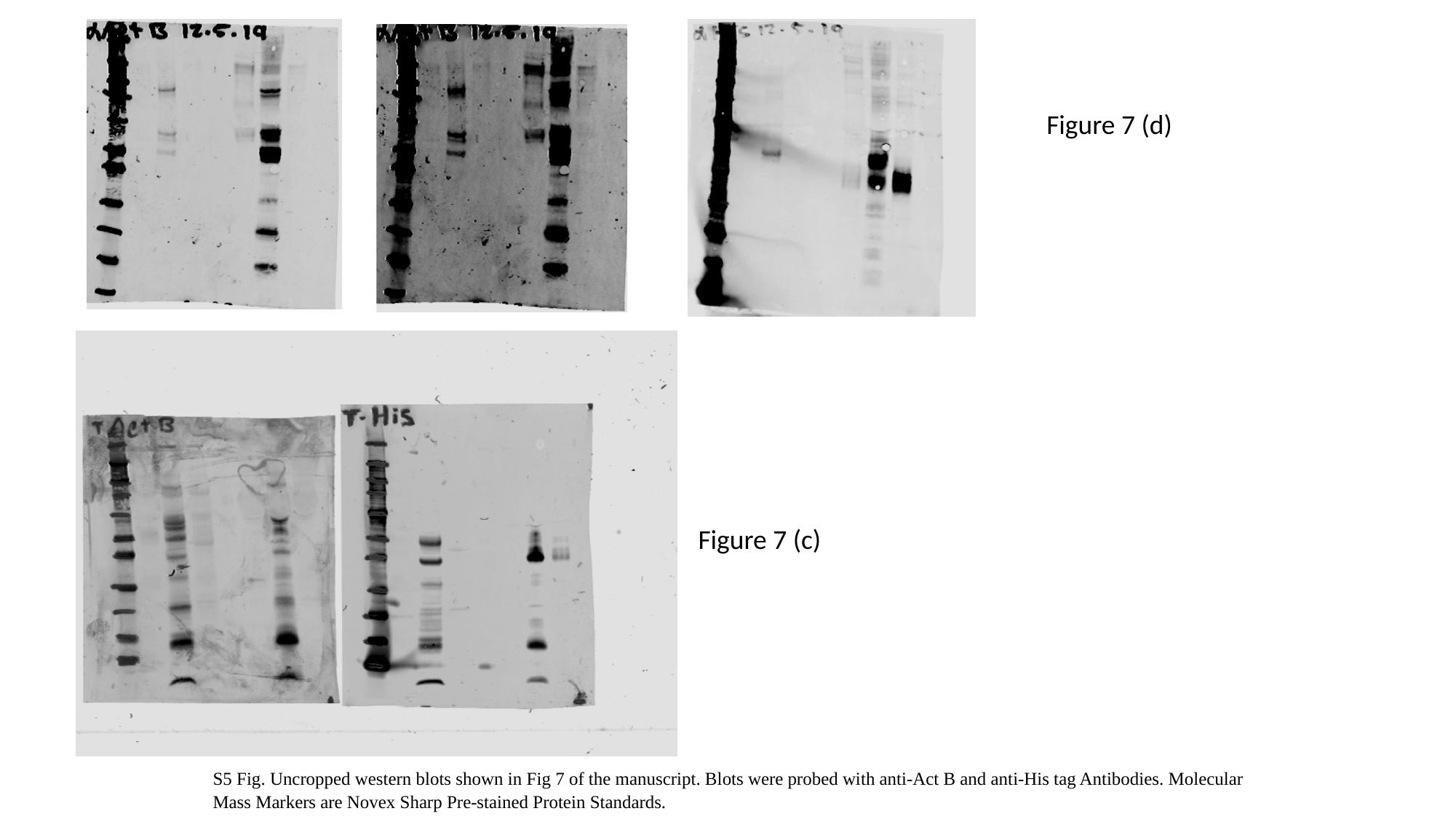

Figure 7 (d)
Figure 7 (c)
S5 Fig. Uncropped western blots shown in Fig 7 of the manuscript. Blots were probed with anti-Act B and anti-His tag Antibodies. Molecular Mass Markers are Novex Sharp Pre-stained Protein Standards.
