# Supplementary material for: Human and mouse activin genes: Divergent expression of activin A protein variants and identification of a novel heparan sulfate-binding domain in activin B
Source: PLoS One. 2020 Feb 19;15(2):e0229254. doi: 10.1371/journal.pone.0229254 (PMC7029874; doi:10.1371/journal.pone.0229254)
Supplement: S1 Table — (DOCX) [file pone.0229254.s006.docx]

S1 Table. Mouse and Human Activin Proteins.

| **Protein** | **Accession^1^** | **Size^2^** | **Signal^3^** | **Pro^3^** | **Ligand^3^** | **Homology^4^** | **Furin Site^5^** |
| --- | --- | --- | --- | --- | --- | --- | --- |
| mINHβA | NP_032406 | 424 | 1-20 | 21-308 | 309-424 | 96% | **RRR**GLE |
| hINHβA | NP_002183 | 426 | 1-20 | 21-310 | 311-426 |  | **RRR**GLE |
|  |  |  |  |  |  |  |  |
| mINHβB | NP_032407 | 411 | 1-28 | 29-296 | 297-411 | 96% | **RKR**GLE |
| hINHβB | NP_002184 | 407 | 1-28 | 29-292 | 293-407 |  | **RKR**GLE |
|  |  |  |  |  |  |  |  |
| mINHβC | NP_034695 | 352 | 1-18 | 19-236 | 237-352 | 76% | **RRR**GID |
| hINHβC | NP_005529 | 352 | 1-18 | 19-236 | 237-352 |  | **HRR**GID |
|  |  |  |  |  |  |  |  |
| mINHβE | NP_032408 | 350 | 1-21 | 22-236 | 237-350 | 82% | **RRR**TPT |
| hINHβE | NP_113667 | 350 | 1-19 | 20-236 | 237-350 |  | **RRR**TPT |
|  |  |  |  |  |  |  |  |

1. NCBI Protein Accession number.

2. Number of amino acids in pre-proprotein.

3. Position of Signal sequence, proregion and active ligand.

4. Percent amino acid identity between full-length Murine and Human proteins.

5. Sequence of Furin cleavage site.
