# Supplementary material for: Human and mouse activin genes: Divergent expression of activin A protein variants and identification of a novel heparan sulfate-binding domain in activin B
Source: PLoS One. 2020 Feb 19;15(2):e0229254. doi: 10.1371/journal.pone.0229254 (PMC7029874; doi:10.1371/journal.pone.0229254)
Supplement: S2 Table — (DOCX) [file pone.0229254.s007.docx]

**S2 Table**. Primers used to amplify Murine INHβA and INHβB transcripts.

| Primer | **Target^1,2^** | **Acession** | **Sequence (5'-3')** | **Region^3^** | **Exon** |
| --- | --- | --- | --- | --- | --- |
| 1 | mINHβA | XM_011244285 | F: AAAGTGTGAAATGGTAATCACTTTC | 3493- 3517 | 1 |
| 2 | mINHβA | XM_011244285 | F: CACCTTTCAGGAGTGAGTAAA | 3997-4017 | 1 |
| 3 | mINHβA | XM_011244285 | F: ACTGGATTAG AATCAAAGCC T | 4588-4605 | 1 |
| 4 | mINHβA | XM_011244285 | F: GCTGGTGTTCAGGGTAAAA ATA | 4753-4774 | 2 |
| 5 | mINHβA | XM_011244285 | F: AGGAGAGTT CACTTCTTTTCATTAAA | 6146-6168 | 2 |
| 6 | mINHβA | XM_011244285 | F: CAATGTGTGAGTGTACAACTAAGTA | 6904-6928 | 2 |
| 7 | mINHβA | XM_011244285 | F: AAAAG CAGGGCCTTT AAAGAA | 7098-7118 | 3 |
| 8 | mINHβA | XM_011244285 | F: GCTT TGGCTGAGAG GATTT | 7154-7172 | 3 |
| 9 | mINHβA | XM_011244285 | F: CTAACTCTCAGCCAGAGATG | 7288-7307 | 4 |
| 10 | mINHβA | XM_011244285 | R: CATCTCTGGCTGAGAGTTAG | 7307-7288 | 4 |
| 11 | mINHβA | XM_011244285 | F: AAGTCACCATCCGTCTATTT | 7654-7673 | 4 |
| 12 | mINHβA | XM_011244285 | R: AAATAGACGGATGGTGACTT | 7673-7654 | 4 |
| 13 | mINHβA | XM_011244285 | F: GAGGAAATG GGCTTAAAGG | 7722-7740 | 4 |
| 14 | mINHβA | XM_011244285 | R: CCTTTAAGCCCATTTCCTC | 7740-7722 | 4 |
| 15 | mINHβA | XM_011244285 | F: GCTGTAAGAAACAGTTCTTT GTC | 8101-8123 | 4 |
| 16 | mINHβA | XM_011244285 | R: GACAAAGAACTGTTTCTTACAGC | 8123-8101 | 4 |
| 17 | mINHβA | XM_011244285 | F: ATTCAA AACATGATTG TGGAGGA | 8382-8404 | 4 |
| 18 | mINHβA | XM_011244285 | R: TCCTCCACAATCATGTTTTGAAT | 8404-8382 | 4 |
|  |  |  |  |  |  |
| 19 | mINHβB | NM_008381 | F: ACATGGACGG GCTGCCCGGT | 1162-1180 | 1 |
| 20 | mINHβB | NM_008381 | F: TCACGGCCCTGCGCAAGC | 1485- 1502 | 1 |
| 21 | mINHβB | NM_008381 | F: ATCATCAGCTTTGCAGAGAC AG | 1601-1622 | 1 |
| 22 | mINHβB | NM_008381 | R: ATGCGATGTCTGCTATCGC | 2040- 2022 | 2 |
| 23 | mINHβB | NM_008381 | R: TTGCCTCTGTCAGGCGCAG | 2407- 2389 | 2 |
|  |  |  |  |  |  |

1. Coding region for mINHβA (XM_011244285): 7146-8420

2. Coding region for mINHβB (NM_008381): 1163-2398

3. Annealing position of primer.
